# The Effect of Sodium Bentonite in the Thermo-Catalytic Reduction of Viscosity of Heavy Oils

**DOI:** 10.3390/molecules28062651

**Published:** 2023-03-15

**Authors:** Zhichao Zhou, Wangyuan Zhang, Tao Yu, Yongfei Li, Alena Struhárová, Marián Matejdes, Michal Slaný, Gang Chen

**Affiliations:** 1 Shaanxi Engineering Research Center of Oil and Gas Field Chemistry, Xi’an Shiyou University, Xi’an 710065, China; 2 Shaanxi Province Key Laboratory of Environmental Pollution Control and Reservoir Protection Technology of Oilfields, Xi’an Shiyou University, Xi’an 710065, China; 3 State Key Laboratory of Petroleum Pollution Control, Xi’an Shiyou University, Xi’an 710065, China; 4 Department of Materials Engineering and Physics, Faculty of Civil Engineering, Slovak University of Technology in Bratislava, Radlinskeho 11, 810 05 Bratislava, Slovakia; 5 Institute of Inorganic Chemistry, Slovak Academy of Sciences, Dúbravská Cesta 9, 845 36 Bratislava, Slovakia; 6 Institute of Construction and Architecture, Slovak Academy of Sciences, Dúbravská Cesta 9, 845 03 Bratislava, Slovakia

**Keywords:** heavy oil, aquathermolysis, synergistic catalysis, catechol-metal complex, bentonite

## Abstract

To study the synergistic catalysis of an ex situ catalyst and in situ clay in the aquathermolysis of heavy oil, in this paper, a series of bentonite-supported catechol-metal complexes were prepared, and the catalytic viscosity reduction performance in the aquathermolysis of heavy oil was investigated. Under the optimized conditions, the viscosity can be reduced by 73%, and the pour point can be lowered by 15.0 °C at most, showing the synergistic catalysis of the ex situ catalyst and in situ clay in this aquathermolytic reaction. Thermogravimetry, physical adsorption-desorption, and scanning electron microscopy were conducted to characterize the thermal stability and microstructure of the ex situ catalyst. The components of the heavy oil before and after the reaction were fully characterized. Six model compounds were used to simulate the aquathermolysis reaction process. In order to study the mechanism of viscosity reduction after the catalytic aquathermolysis reaction, the compounds were analyzed by GC-MS. It is believed that these results will be beneficial in the future for related research in this field.

## 1. Introduction

With the continuation of oil production, the world’s light oil reserves are constantly shrinking. Global heavy oil reserves are still abundant; therefore, increasing light oil production has very important economic value. The exploitation of heavy oil is mainly hampered by its high viscosity and high pour point [1]. Due to these obstacles, the traditional exploitation methods are no longer applicable [2,3,4]. In order to improve its economic benefits, many technologies, such as chemical flooding, thermal flooding, and steam-assisted gravity flooding are used. Nevertheless, the utilization of these technologies can be very complicated for the following reasons. The viscosity reduction effect of chemical flooding agents varies greatly in different oil regions [5]. Thermal flooding has the widest range of applications, but the oil viscosity remains higher after oil production [6,7]. Steam-assisted gravity flooding is limited by the various factors of layers of soil; therefore, the technique is still at the theoretical stage [8,9]. All the above technologies reduce the viscosity of heavy oil by physically reducing its viscosity for extraction purposes. Therefore, they cannot fundamentally solve the problem of the high viscosity of heavy oil, nor can they target the components affecting the high viscosity, such as resins and asphaltenes. Despite high levels of upgrading, the problem of high viscosity in heavy oil has not yet been solved effectively.

In traditional thermal recovery methods, during the process, the introduced high-temperature steam acts as a heat source to reduce the viscosity of the heavy oil contact layer in order to improve its fluidity, requiring increased energy input [10]. When the temperature is lowered, the viscosity of heavy oil returns to its previous value. During the heat transfer process, the heavy oil is under high-temperature conditions; the large molecules are thermally cracked into smaller-sized lighter hydrocarbon molecules, which, in turn, reduce the viscosity of the heavy oil [11,12]. However, the degree of cracking is very low, and it is difficult to achieve the desired effect without the presence of a catalyst [10]. Nowadays, the catalysts used for the aquathermolysis of heavy oil can be divided into six categories: water-soluble catalysts, oil-soluble catalysts, amphiphilic catalysts, minerals and zeolites, solid superacids, and dispersed nanoparticles [13,14,15,16]. The utilization of catalysts improved the viscosity reduction rate of heavy oil to varying extents, achieving satisfactory results. Chen et al. [17] studied the transition metal catalysts used for the low-temperature cracking catalysis of heavy oil, the catalytic effect of which is most efficient at 180 °C. Suwaid et al. [18] prepared oil-soluble transition metal-based catalysts (Fe, Co, Ni) to catalyze the hydrothermal decomposition of heavy oil at 300 °C. This approach enables high catalytic performance by simultaneously promoting the thermal decomposition and hydrogenation reaction of heavy components (rubber grease, asphaltenes, polycyclic aromatic hydrocarbons, long-chain alkanes, etc.). Li et al. [19] catalyzed the hydrothermal cracking of heavy oil using a ZrO_2_-TiO_2_ catalyst modified with CTAB, achieving a 66.3% heavy oil viscosity reduction rate at 180 °C. The effect of alkyl chain length (C2, C4, C6, C8, C10, and C12), the counterion charge (chloride, thiocyanate, and tetrafluoroborate) of the ionic liquid, and skeletal group types (imidazolium, pyridinium, and thiazolium) on the catalysis of Mexican heavy oil and Canadian and Venezuelan pitch was investigated by Deepa et al. [20]. A nanocatalyst for the low-temperature aquathermolysis of heavy oil was prepared by Li et al. [21], achieving a 99.7% viscosity reduction rate. However, the mentioned reports are solely focused on the catalytic effect between the catalyst and the heavy oil; meanwhile, the interplay between the catalyst and the reservoir has been ignored. Studies show that the clay minerals in the reservoir can promote oil reserves from the source rock, while clay minerals mainly promote the conversion of kerogen [22,23]. Due to the properties of clay minerals, they readily undergo ion exchange and are able to act as catalyst carriers [24]. Transition metal catalysts that are introduced into the reservoir will interact with the various mineral components found in the reservoir, forming new catalysts, which will increase the catalytic effect of aquathermolysis, while the viscosity of crude oil will decrease due to the increased hydrocarbon content. Therefore, it is of great significance to study clay-supported catalysts in order to enhance the conversion efficiency of crude oil into smaller-sized hydrocarbon molecules. Certain progress in the field of heavy oil aquathermolysis was achieved by Chen et al. [25,26,27,28,29]. Transition metal ions were employed as a catalyst for the heavy oil aquathermolysis, achieving good viscosity reduction effects. Song et al. [29] used cobalt tartrate as a catalyst, obtaining an improved viscosity reduction rate. The reduction of heavy oil viscosity at low temperatures was demonstrated by using ferric citrate as a catalyst [30]. An 84.2% decrease in viscosity was reached by Hao et al. [31] by employing a series of Mannich base-transition metal coordination complexes.

In this study, the physicochemical conditions of the catalytic decomposition of heavy oil by aquathermolysis were studied. On the basis of previous results, the effect of various minerals on catalysis was investigated. To simulate the catalysis of heavy oil, a combination of a clay mineral with the most optimal properties (sodium bentonite) and a transition metal complex with a catechol ligand was used. TGA/DSC, GC, and GC-MS methods were used to study the ongoing changes during heavy oil aquathermolysis. In addition, commonly used compounds were selected to simulate the material changes of heavy crude oil, and GC-MS was used to determine the individual fractions.

## 2. Experimental

### 2.1. Materials

The heavy oil, L8401, used in the experiments was obtained from the Henan oilfield. The aquathermolysis reaction was conducted in a magnetic miniature high-pressure reactor (WCGF-100ML, volume 1/2 100 mL), equipped with a stirrer manufactured by Xi’an Taikang Biological Technology Co., Ltd. (Xi´an, China). Sodium bentonite was purchased from the Nanyang Haofa Bentonite Co., Ltd. The main components of the clay are SiO_2_, Al_2_O_3_, and Na_2_O. Nonylphenol (guaranteed reagent), benzothiophene (purity over 97%), and thiophene (purity over 99%) were purchased from Shanghai Macleans Biochemical Technology Co., Ltd. (Shanghai, China). Quinoline (analytical reagent) was purchased from Chengdu Kelon Snow Products Co., Ltd. (Chengdu, China). Phenol (analytical reagent) was purchased from Tianjin Beilian Fine Chemicals Development Co., Ltd. (Tianjin, China). Pyridine (analytical reagent) was purchased from Tianjin Fuyu Fine Chemical Co., Ltd. (Tianjin, China).

### 2.2. Determination of Physical Parameters

The density of crude oil was determined according to GB/T 1884-2000. At 30 °C, the density of L8401 was 0.961 g/cm^3^. The pour point was measured according to SY/T 2541-2009. The pour point of L8401 was 24.0 °C.

### 2.3. Initial Crude Oil Reaction, Product Separation, and Analysis

First, 30 g of heavy oil and 9 g of distilled water were added to the reactor (100 mL) for high-temperature experiment. The sample containing oil and water was marked as Oil-water (OW). After the reaction was completed, the heating was stopped (after 6 h of reaction time), then the reaction mixture was cooled down to room temperature and poured into a measuring cup. The measuring cup containing the reaction mixture was placed in a water bath and heated at 70 °C for 2 h to promote the separation of oil and water. The separated oil phase was used for testing and analysis. The aqueous phase was removed with a rotary evaporator, dissolved in absolute ethanol, and analyzed for dissolved substances.

In the same way as previously described, a mixture containing 30 g of heavy oil, 9 g of distilled water, and 0.15 g of sodium bentonite (SB) was prepared. The sample that was taken after the reaction was marked as Oil-water-SB, abbreviated as OWSB. An OW sample containing only catechol chromium was marked as Oil-water-C-Cr, then abbreviated as OWCCr. The sample that was catalyzed by chromium catechol, supported by sodium bentonite, was marked as Oil-water-SBC-Cr, abbreviated as OWSBCCr. The oil obtained from the reaction catalyzed by chromium catechol, supported by sodium bentonite, and 3 g of alcohol was marked as Oil-water-SBC-Cr-ethanol, abbreviated as OWSBCCrE.

In addition, experiments with model compounds under different reaction conditions were also carried out. Phenol was dissolved in n-hexane and other model compounds were dissolved in benzene. Then, 10 g of the model compound, 3 g of water, 0.05 g of sodium bentonite or bentonite-supported chromium catechol, and 1 g of ethanol were weighted to simulate the above hydrothermal cracking reaction of heavy oil. The final reaction solution was centrifuged at 4000× *g* rpm for 30 min; afterward, only the sample taken from the upper layer was analyzed.

### 2.4. Catalyst Preparation

**Figure 1:**
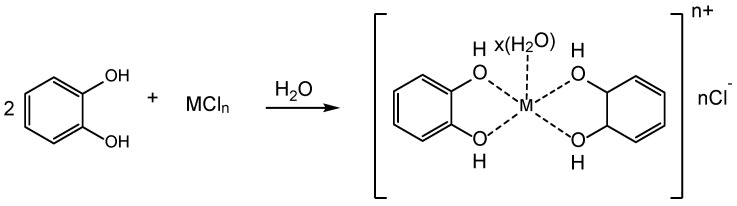
The preparation process of the catechol metal complex (M = Cr, Mn, Fe, Co, Ni, Cu, Zn).

**Figure 2:**
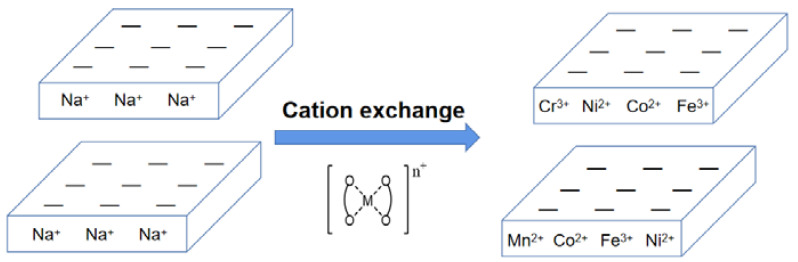
The interaction of the catechol chromium complex and bentonite via cation exchange (M = Cr, Mn, Fe, Co, Ni, Cu, Zn).

Defined amounts of chromium chloride and catechol were weighed at a molar ratio of 1:2 and dissolved in water and ethanol, respectively. After complete dissolution, the catechol solution was slowly dropped into the chromium chloride solution and stirred at 70 °C for 4 h. The reaction between the catechol and metal complex is shown in Figure 1. Subsequently, sodium bentonite was added, according to its minimum ion exchange capacity. The sodium bentonite was stirred at 70 °C for 4 h. The synthesis of sodium bentonite supported with catechol chromium is shown in Figure 2. After cooling down to room temperature, the mixture was centrifuged, washed until it was colorless, and dried at 70 °C. The powdered sodium bentonite-supported metal complex was marked as SBCCr.

### 2.5. Viscosity Evaluation

The oil removed from the reaction kettle was left aside for 4 hours and heated to 70 °C for 2 h. The viscosity of the crude oil was measured according to the standard, ASTM D97-96 [32].

### 2.6. Differential Scanning Calorimetry Analysis (DSC)

The wax precipitation point and the amount of crude oil were measured according to SY/T 0545-2012. The different scanning calorimetry (DSC) analysis of heavy oil was performed on a Mettler-Toledo DSC822e DSC (Columbus, OH, USA) in a nitrogen atmosphere at a flow rate of 20 mL/min and at a scan rate of 10 °C/min between −20 and 50 °C.

### 2.7. BET-N_2_ Adsorption

The specific surface areas (SSA) of the samples were determined by nitrogen adsorption using a Quantachrome Autosorb iQ apparatus. Before each measurement, the samples were degassed for 12 h at 105 °C. The specific surface area calculation was based on the Brunauer Emmett Teller (BET) formalism.

### 2.8. Gas Chromatography (GC)

Saturated hydrocarbons were obtained via the separation of crude oil components. The distribution of saturated hydrocarbons, in terms of their numbers of structural carbons, was analyzed in the final oil sample under different reaction conditions via gas chromatography. The injector temperature was held at 240 °C, the oven temperature was at 50 °C, and the detector temperature was at 280 °C.

### 2.9. TGA Analysis

Thermogravimetric analysis (METTLER-TOLEDO modul Star TGA/DSC-1, Columbus, OH, USA) was used to evaluate the distribution of the carbon numbers of crude oil within different temperature ranges. The oil samples were heated from 25 °C to 1000 °C under a nitrogen atmosphere, while the heating rate was kept at 10 °C/min.

### 2.10. GC-MS

The composition analysis of the model compound was measured via GC-MS using a 7890A-5975C (Santa Clara, CA, USA), with hydrogen as a carrier gas, keeping the flow rate at 25 mL/min. The composition analysis of the samples was identified by using the DRS compound database. Further measurement details are described elsewhere by K. Chao [33].

### 2.11. Elemental Analysis and SARA Analysis

The elemental composition (C, H, N, and S) of the initial and heavy oils was measured with an Elementar Vario EL cube (Langenselbold, Germany). Determination of the four components of petroleum asphaltene was performed according to standard NB/SH/T0509-2010.

### 2.12. Scanning Electron Microscopy (SEM)

Scanning electron microscope (SEM) Vega 3, Tescan, (VEGA 3 SBU, TESCAN, Brno, Czech Republic) equipped with SE and BSE detectors was used for observing samples morphology at a voltage of 30 kV. The samples were coated with gold.

## 3. Results and Discussion

### 3.1. Thermogravimetric Analysis of the Catalyst

**Figure 3:**
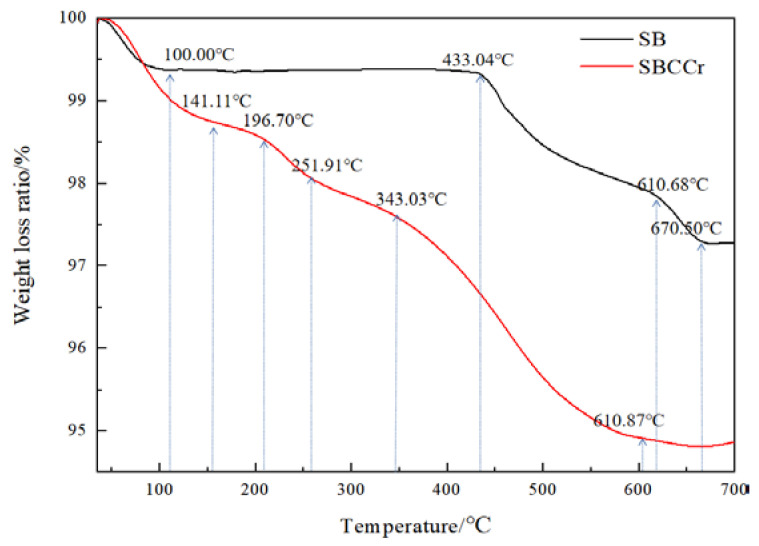
The TGA curve of sodium bentonite with and without catechol chromium catalyst.

In order to extend the usage and the lifetime, the thermal stability of the catalyst plays a crucial role. The weight loss curves of sodium bentonite with and without chromium catechol are shown in Figure 3. It can be seen that the weight loss curves of sodium bentonite with and without chromium catechol follow completely different paths. The more pronounced weight loss of bentonite with catechol chromium at lower temperatures up to 200 °C can be described as a loss of water that is physisorbed on the surface. The weight loss of pure sodium bentonite is at 200–450 °C and is nearly constant, confirming the better thermal stability of sodium bentonite. The small loss can be described as the dehydration and condensation of sodium bentonite surface hydroxyl groups. In the case of bentonite supported with catechol chromium, the weight loss is in the range of 200–450 °C and is much more prominent; it is mainly caused by the thermal degradation of catechol chromium. The weight loss between 610 and 670 °C is related to dehydroxylation. Based on the weight loss curve comparison between sodium bentonite with and without chromium catechol shows that the chromium catechol was successfully intercalated into the interlayer space of the clay mineral.

### 3.2. Adsorption-Desorption Curves

**Figure 4:**
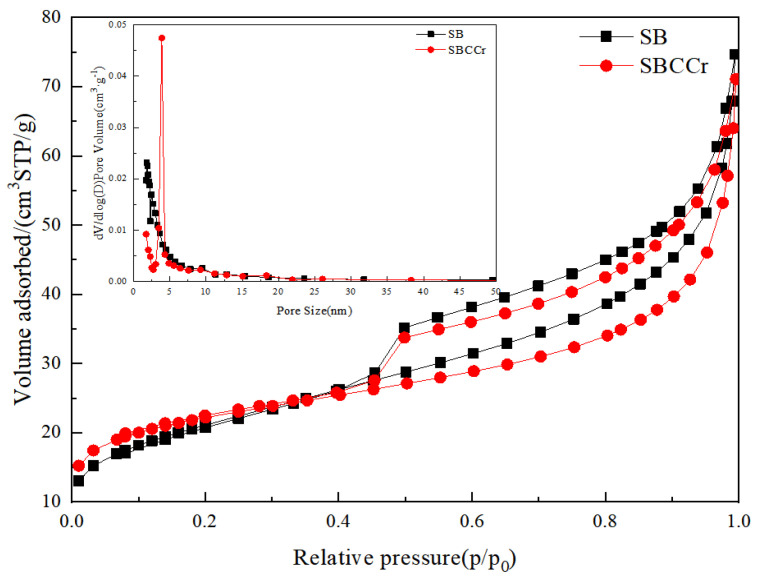
Adsorption/desorption isotherms and pore size distribution of SB and SBCCr.

The N_2_ adsorption and desorption curves of sodium bentonite with and without catechol chromium and the corresponding pore size distribution diagrams are shown in Figure 4. According to the IUPAC classification, the N_2_ adsorption and desorption curves of sodium bentonite exhibit a type-IV isotherm, indicating the presence of mesopores. The adsorption isotherms of SB and SBCCr contain hysteresis loops, reflecting the increased adsorption capacity with increased pressure, at the same time indicating the presence of large pores [32]. The adsorption process of SBCCr is divided into three stages: when 0 < *p*/*p*_0_ < 0.4, the nitrogen pressure is low, and the sample adsorption isotherm increases slowly; in this region, nitrogen is on the surface and is arranged in a monolayer, 0.4 < *p*/*p*_0_ < 0.8, the nitrogen adsorption rate is accelerated, the adsorption isotherm rises rapidly and, consequently, a hysteresis loop appears. Nitrogen is now adsorbed in a multilayer fashion; at 0.8 < *p*/*p*_0_, the nitrogen adsorption rate increases sharply. Close to the saturated vapor pressure, the adsorption saturation phenomenon occurs, and the sample is found in the capillary condensation stage. The adsorption and desorption curve of the H3 hysteresis loop rises slowly, and the adsorption curve increases rapidly when *p*/*p*_0_ approaches 1, reflecting the evolution from mesopores to macropores of the sample.

According to the pore size distribution curve shown in the inset of Figure 4, it can be seen that in the sample of sodium bentonite that has undergone ion exchange, a strong spike appeared at 3 nm, indicating that the 2 nm pores in sodium bentonite are replaced with the larger pores found in the SBCCr sample. The BET parameters before and after ion exchange are shown in Table 1. It was found that there is no significant difference in the pore structure parameters of SB and SBCCr, indicating the preservation of its textural properties.

### 3.3. SEM Analysis

**Figure 5:**
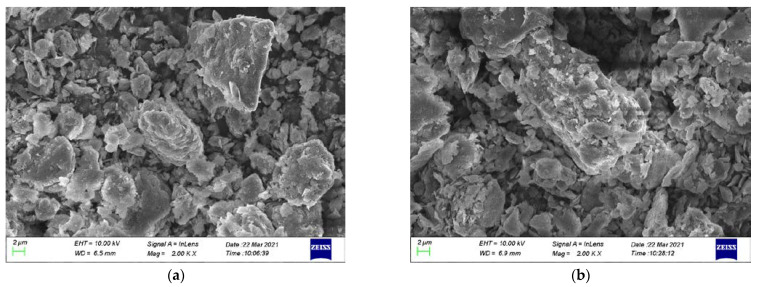
SEM micrographs of SB and SBCCr. (**a**) Micromorphology of sodium bentonite. (**b**) Micromorphology of sodium bentonite loaded with chromium catechol.

According to the SEM measurements (Figure 5), sodium bentonite is mostly made up of particles with different shapes and sizes. Comparing SB and SBCCr, the size of the catalyst does not change significantly, and some particles are dispersed into smaller particles.

### 3.4. Optimal Physical and Chemical Conditions

**Figure 6:**
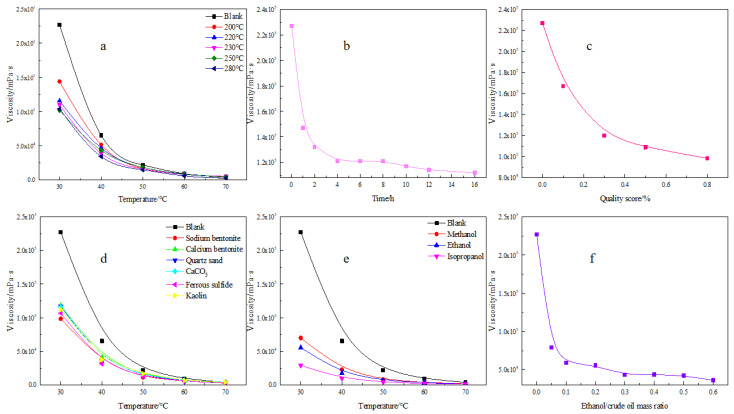
Viscosity reduction under different reaction conditions depending on. (**a**) temperature; (**b**) time; (**c**) dosage; (**d**) minerals composition; (**e**) solvents and (**f**) ethanol.

The aquathermolysis of heavy oil requires a high temperature. Firstly, the influence of temperature on the aquathermolysis reaction of L8401 heavy oil was investigated. The L8401 heavy oil was reacted at 200, 220, 230, 250, and 280 °C for 12 h. The blank control is represented by the oil sample, without any treatment at elevated temperatures. As shown in Figure 6a, the drop in viscosity of L8401 heavy oil was found to be greatest at a reaction temperature of 250 °C. Compared with 250 °C, at 280 °C, the viscosity of the heavy oil did not change significantly, and coke formation was identified at the bottom of the reactor. Therefore, 250 °C was selected as the subsequent reaction temperature. As shown in Figure 6b, as the reaction time proceeds, the viscosity decreases; after 4 h, it remains almost unchanged, hence, the reaction time for the subsequent reactions was set to 4 h. Figure 6c shows that as the dosage of sodium bentonite increases, the viscosity is reduced. Considering the economic aspects, the dosage of sodium bentonite for the following experiments was selected to be 0.5%. The effect of an admixture of 0.5% sodium bentonite, calcium bentonite, quartz sand, ferrous sulfide, kaolin, and calcium carbonate on the viscosity drop of L8401 heavy oil, reacted at 250 °C for 4 h, is shown in Figure 6d. The minerals used proved to have a good catalytic performance in terms of viscosity reduction. Among them, sodium bentonite showed the best results, followed by ferrous sulfide, while the worst performance can be attributed to calcium carbonate; hence, for the following experiments, sodium bentonite was selected. For the aquathermolysis reaction, hydrogen donors such as methanol, ethanol, and isopropanol were evaluated as well. As shown in Figure 6e, all of them were able to reduce the viscosity. Despite the fact that isopropanol has the best performance, it has obvious solvent dilution effects and a higher price; therefore, for the subsequent experiments, ethanol was selected. Finally, the dosage of ethanol was optimized as well. As shown in Figure 6f, as the ethanol/oil mass ratio increases, the viscosity of the heavy oil continues to decrease until the ethanol/oil mass ratio reaches 0.1; therefore, this value was established for subsequent experiments.

### 3.5. Viscosity Analysis of Heavy Oil Catalyzed by Different Catalysts

**Figure 7:**
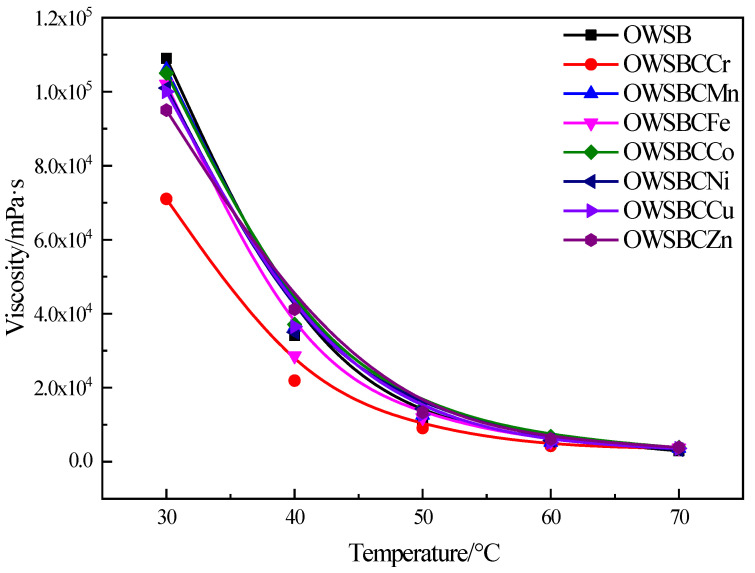
The effect of different metal-loaded sodium bentonite samples on viscosity reduction.

As shown in Figure 7, the effect of the sodium bentonite-supported transition metal complexes with respect to viscosity reduction was evaluated. Based on the catalytic activity of sodium bentonite (52% viscosity reduction at 30 °C), as different metal complexes were supported, the viscosity reduction effect was further improved, indicating that the ex situ catalyst has a synergistic catalytic effect with the minerals present in the reservoir. Compared to the blank, the highest catalytic activity can be attributed to sodium bentonite-supported chromium catechol, with a 68% viscosity reduction at 30 °C. In other words, compared to using clay as a catalyst alone, the viscosity is reduced by a further 36%. The obtained results are closely related to the structure of the catalyst, as the ligand plays an important role in activating and stabilizing the core metal ion. Catalytic activity is also correlated to the occupancy of the d-orbitals. The d-orbitals of the chromium catechol complex are half-occupied, allowing unoccupied orbitals to be involved in the catalytic process. From the catalysis point of view, chromium has a considerable advantage over other transition metals, showing limited catalytic activity.

**Figure 8:**
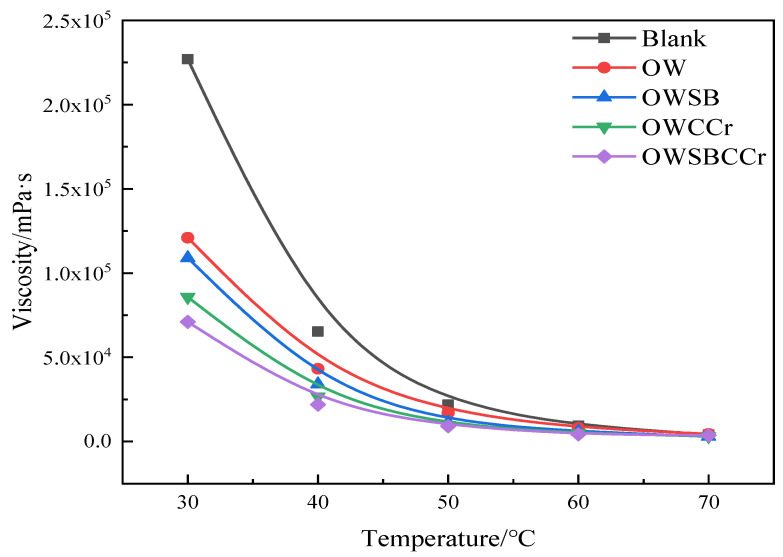
Effect of the ex situ catalyst and in situ bentonite on the reduction in heavy oil viscosity.

As illustrated in Figure 8, the heavy oil viscosity reduction after applying only sodium bentonite was 52%; after adding catechol chromium, the reduction reached 62%. When bentonite was combined with catechol chromium, the viscosity reduction was 68%. This is 35% more than in the case of the Oil-water-SB sample employing only bentonite and 21% higher in the case of the Oil-water-C-Cr sample containing only catechol chromium. These findings indicate the presence of a catalytic synergistic effect between catechol chromium and bentonite, having a significant impact on the reduction in heavy oil viscosity.

**Figure 9:**
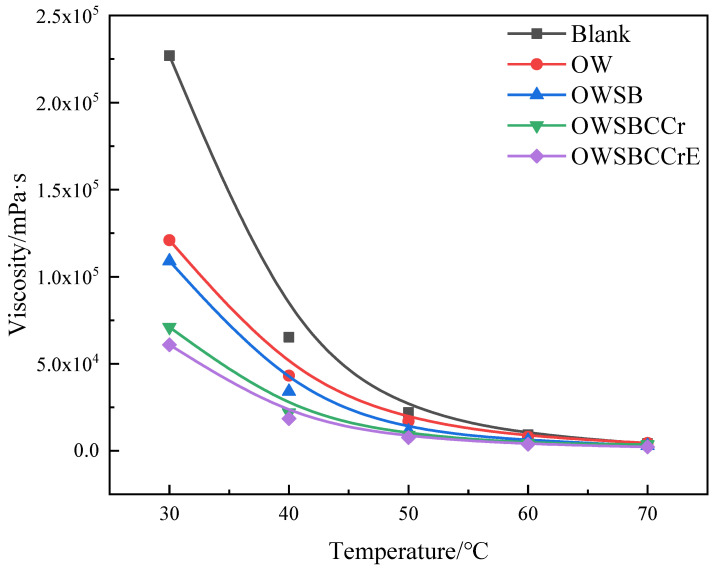
The viscosity of L8401 after reactions under different conditions.

As mentioned above, the effect of the hydrogen donor on viscosity reduction was investigated as well. The viscosity reduction in the Oil-water, Oil-water-SB, Oil-water-SBC-Cr, and Oil-water-SBC-Cr-ethanol samples reached 46%, 52%, 68%, and 73%, respectively (Figure 9). This observation clearly shows that bentonite has a certain catalytic activity. When using NBC-Cr, the viscosity reduction was 16% higher than that of Oil-water-SB, showing the synergistic catalysis of the ex situ catalyst and in situ clay in heavy oil aquathermolysis. After ethanol addition, the viscosity reduction was 5% higher than that of Oil-water-SBC-Cr, indicating the hydrogen donor ability of ethanol to enhance the viscosity reduction. 

Compared with the optimal temperature recorded by Suwaid et al. [18], which was 50 °C lower, the reaction time was shortened by 20 h, and the viscosity was reduced by 23.7%. Compared with the settings reported by Chao et al., [33] the temperature was lowered by 30 °C, the reaction time was shortened by 20 h, and the viscosity was reduced by 22.5%.

Clay minerals contain a large number of acid sites, which are able to produce silicic acid during the reaction process at high temperatures. The interaction between silicic acid and water will produce a large amount of active hydrogen, which participates in the alkyl chain-breaking reaction. Consequently, the formed short-chain hydrocarbons directly contribute to heavy oil viscosity reduction. The presence of clay minerals also promotes the oxidation of ethanol, which was proposed in the study by Chen et al. [34].

### 3.6. Pour Point Depression

The pour point of the blank oil sample is 24 °C, and after the aquathermolysis reaction of heavy oil is further depressed below this temperature (Table 2). When using sodium bentonite as a catalyst, the pour point does not change very much. When using sodium bentonite supported with an ex situ catalyst, the pour point was depressed significantly by about 15.0 °C. The presence of the hydrogen donor had a negligible effect on the pour point. Under optimized conditions, the pour point was depressed by 3.0 °C, 5.0 °C, 10.0 °C, 15.0 °C, and 15.0 °C, respectively, showing the synergistic catalysis effect of the ex situ catalyst and in situ clay during the aquathermolysis of heavy oil.

### 3.7. Catalyst Stability Evaluation

After the reaction, the final reaction mixture was centrifuged, then the catalyst was collected and reused. It can be seen from Table 3 that after the catalyst was reused 4 times, the viscosity and pour point of the heavy oil did not change significantly. It is believed that the confirmed catalysts’ stability makes them suitable for practical use in heavy oil exploration.

### 3.8. Viscosity Stability Evaluation

**Figure 10:**
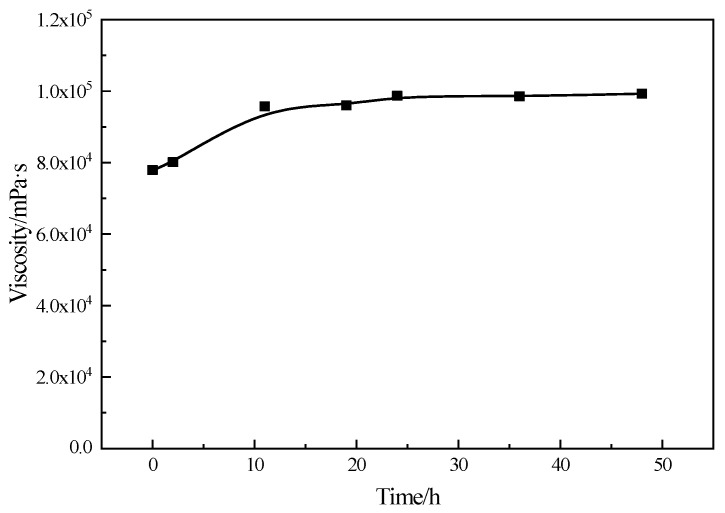
The change of viscosity with time (OWSBCCr).

After the reaction, the viscosity of the heavy oil was measured at 30 °C with respect to time. The viscosity of heavy oil increases rapidly within 10 h; after this time, the viscosity basically remains unchanged (Figure 10). This behavior indicates that the heavy oil heteroatom-containing groups re-establish the intermolecular forces such as hydrogen bonds. The re-entanglement of the resin causes the viscosity to rise, then it remains the same after 10 h.

### 3.9. TGA/DTG Analysis

**Figure 11:**
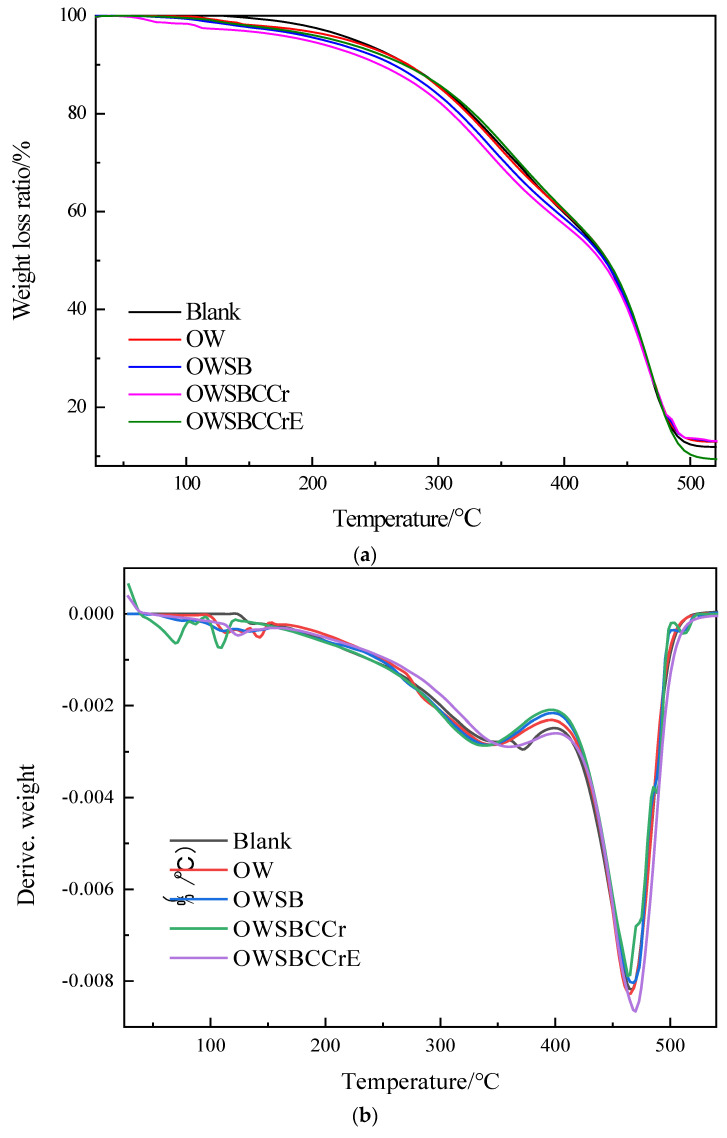
L8401 heavy oil under different reaction conditions: (**a**) TGA curve of the different oil samples, (**b**) DTG of different oil samples).

The TGA analysis of the heavy oil before and after catalytic aquathermolysis under different reaction conditions is shown in Figure 11. Only a slight weight loss ratio can be seen at 28~150 °C. However, this may indicate that under different treatment conditions, the light oil content increased after the reaction. The weight loss ratios at 150–350 °C and 350–450 °C were already much higher. The weight loss rates above 450 °C were 29.36%, 28.80%, 28.21%, 27.20%, and 33.12%, respectively. The oil sample produced from the Oil-water-SBC-Cr-ethanol sample had the highest light oil component content and the minimum residue.

### 3.10. Elemental Analysis

Elemental analysis of the heavy oil before and after catalytic aquathermolysis under different reaction conditions is shown in Table 4. The total proportion of C, H, N, and S decreased after the oil-water reaction, especially the C content, which decreased sharply with a concurrent H/C ratio increase, indicating the increased saturation caused by hydrogenation. The sulfur content decreased, indicating that an external catalyst is also suitable for use in the heavy oil desulfurization process. The C/H ratio, S, and N contents of the OWSBCCrE sample decreased after the reaction, indicating that the heavy oil heteroatoms were also effectively removed.

### 3.11. Differential Scanning Calorimetry (DSC)

**Figure 12:**
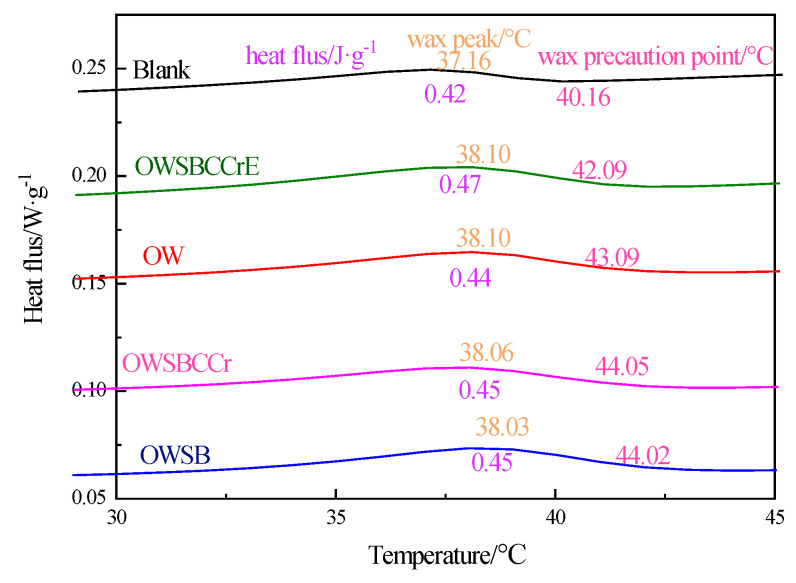
The DSC of heavy oil that has reacted under different conditions.

The precipitation and dissolution of wax in crude oil are greatly influenced by temperature. When the temperature drops to a certain threshold, the wax separates from the crude oil, resulting in a reduction in the oil’s fluidity. DSC was used to study the crystallization of wax in different samples of heavy crude oil. Figure 12 shows the wax precipitation point, wax precipitation peak and the volume of wax precipitated in heavy crude oil after the reaction under different reaction conditions. The amount of precipitated wax in the different oil samples was calculated according to the following formula:

$$w=\frac{\int_{t_{0}}^{-20}Qd_{Q}}{\bar{Q}}$$

where *d_Q_* is the heat released from the wax crystallization in the oil samples at temperature t~(t + dt), J/g; $\bar{Q}$ is the average crystallization heat of crude oil, the value of which is 210 J/g.

Compared to the blank, the wax precipitation point and the wax peak of the sample shifted to the right after the reaction. The reason for this may be that the structure of resin and asphaltene in the heavy oil had been disrupted to different degrees, which weakened the dispersion effect of the wax from crude oil so that the wax can accumulate and increase its volume rapidly [34].

### 3.12. Gas Chromatography (GC) Analysis of the Saturated HC

**Figure 13:**
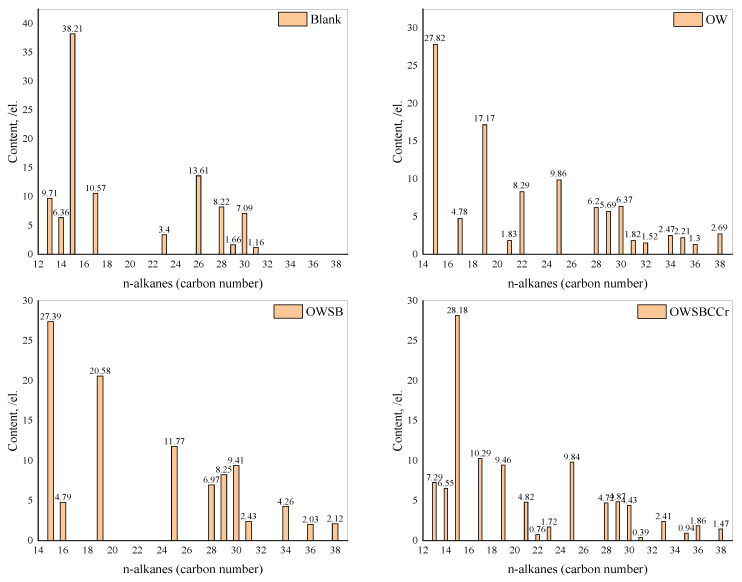

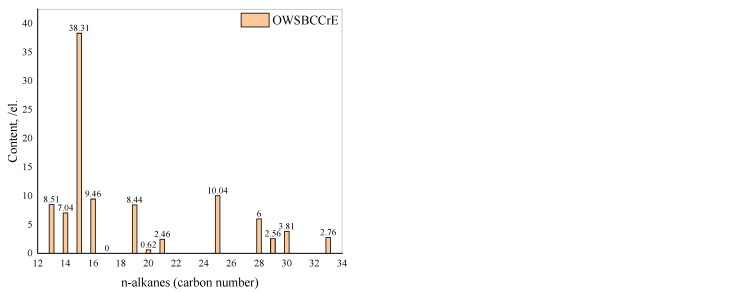
Distributions of saturated hydrocarbons after reaction under different conditions.

In order to separate the saturated hydrocarbons, the oil samples were separated via a four-component separation method and dissolved in n-hexane for the GC analysis [18]. As shown in Figure 13, compared to the blank, the saturated hydrocarbons in the oil-water sample are located in a higher carbon number range of 25–38, while the oil-water-SB sample has a high C19 content. The carbon number of the OWSBCCr sample is distributed over a wide range, and the carbon number of the OWSBCCrE sample is mainly found between C12 and C25.

## 4. Mechanism

### 4.1. Catalytic Aquathermolysis of Model Compounds

The catalytic aquathermolysis reaction of heavy oil mainly involves the reaction of resin and asphaltenes, which are macromolecules containing five- and six-membered rings, with heteroatoms and alkyl branches. According to the chemical structure, a series of model compounds, which can represent its special chemical structure, was selected to study the catalytic aquathermolysis in order to explore the viscosity reduction mechanism. The selected model compounds are phenol, thiophene, nonylphenol, quinoline, benzothiophene, and pyridine. According to the model compound and reaction conditions, the samples are denoted as X-W, X-WSB, X-WSBCCr, and X-WSBCCrE (where X denotes the model compound). The aquathermolysis reactions of model compounds were carried out according to the procedure in Section 3.3 and the liquid phase was separated for the subsequent GC-MS analysis. 

**Scheme 1:**
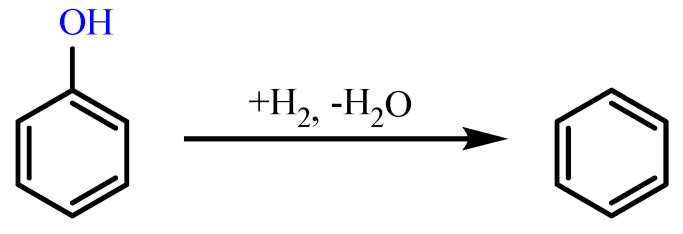
Phenol’s dehydroxylation.

Table 5 shows the GC result of the phenol sample after the reaction under different conditions. Under the optimized reaction conditions, a new compound, benzene, was detected as the product of a reaction between phenol and water indicating the phenol’s dehydroxylation. The reaction is shown in Scheme 1.

**Scheme 2:**
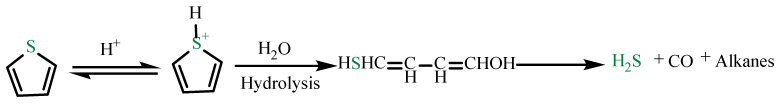
Reaction process of thiophene.

The result of the reaction of thiophene under different reaction conditions is summarized in Table 6. In the reaction, the phenol was detected and formed by an oxidation–reduction reaction between the benzene, H_2_, and CO in the reaction. The CO may have originated from the thiophene, indicating that the thiophene reacted. However, the chromatogram shows no other thiophene derivatives, suggesting that the thiophene generates smaller gas molecules that escape from the liquid phase. The production of toluene may be caused by the attack of a small alkane molecule, formed during the reaction on the benzene ring. The reaction process of thiophene is shown in Scheme 2.

**Scheme 3:**
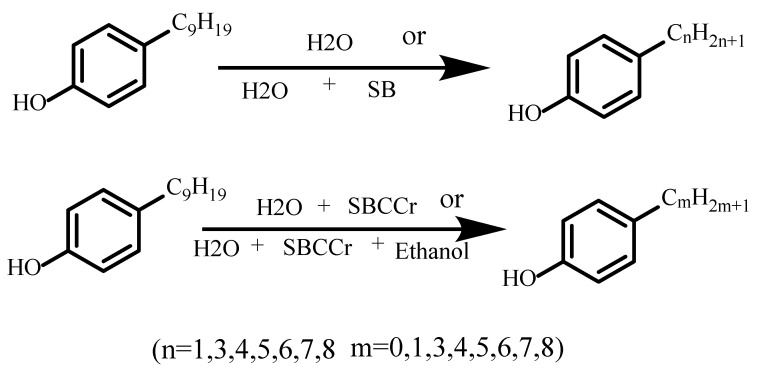
Reaction process of nonylphenol.

Table 7 shows the GC result of the nonylphenol after the reaction under different reaction conditions. Due to the large molecular weight and polarity of nonylphenol, the differences between products are very small, so that a compact range of multiple peaks appeared in the gas chromatogram. The difference in nonylphenol under different reaction conditions is quite obvious. The reaction results of nonylphenol-W and nonylphenol-WSB samples are similar, and the new products are alkylphenol with different alkyl chain lengths. Similarities can be also seen between nonylphenol-WSBCCr and nonylphenol-WSBCCrE. The new products are alkylphenols with different alkyl chain lengths and phenol, indicating that the addition of external catalysts and hydrogen donors makes the reaction easier. The reaction process is shown in Scheme 3.

**Scheme 4:**
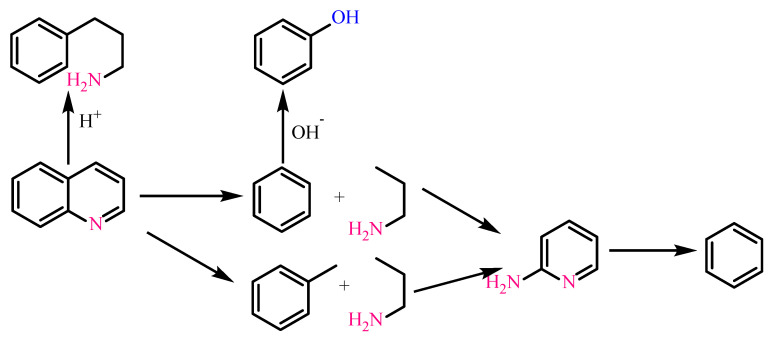
Reaction process of quinoline.

The GC result for quinoline is summarized in Table 8. The reaction of the Oil-water sample is severely limited at high temperatures and the reaction yield is very low. In the nitrogen-containing ring, the C-C and C-N bonds are predominantly broken to form 3-phenyl-1-propylamine, benzene, propylamine, and toluene. Benzene generates phenol when under attack from the hydroxyl group. The catalyst (SB or SBCCr) was added during the reaction to promote a reaction between propylamine and also to promote pyridine production. Upon the addition of the hydrogen donor, only toluene was detected, indicating that the presence of the hydrogen donor promotes the conversion of quinoline to toluene and inhibits the production of pyridine. The reaction process is shown in Scheme 4.

**Scheme 5:**
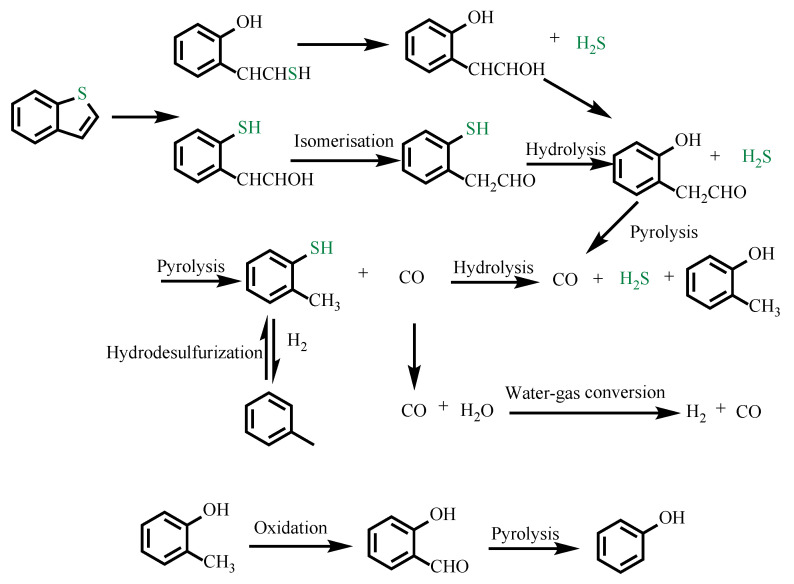
Reaction process of benzotiophene.

Table 9 shows the gas chromatogram of benzothiophene after a reaction under different reaction conditions. In the benzothiophene-WSB and benzothiophene-WSBCCrE samples, the product types after the reaction were the same as for the benzothiophene-W and benzothiophene-WSBCCr samples. The addition of water and a hydrogen donor results in a new substance, 2-mercaptotoluene. The production of phenol may be related to the operation process. The desulfurization reaction occurred mainly during the benzothiophene reaction, while the benzene ring remained preserved. The reaction process is shown in Scheme 5.

No new substances were detected under the different reaction conditions when pyridine (Table 10) was used, indicating that pyridine is chemically stable under the given conditions.

### 4.2. Viscosity Reduction Mechanism

**Figure 14:**
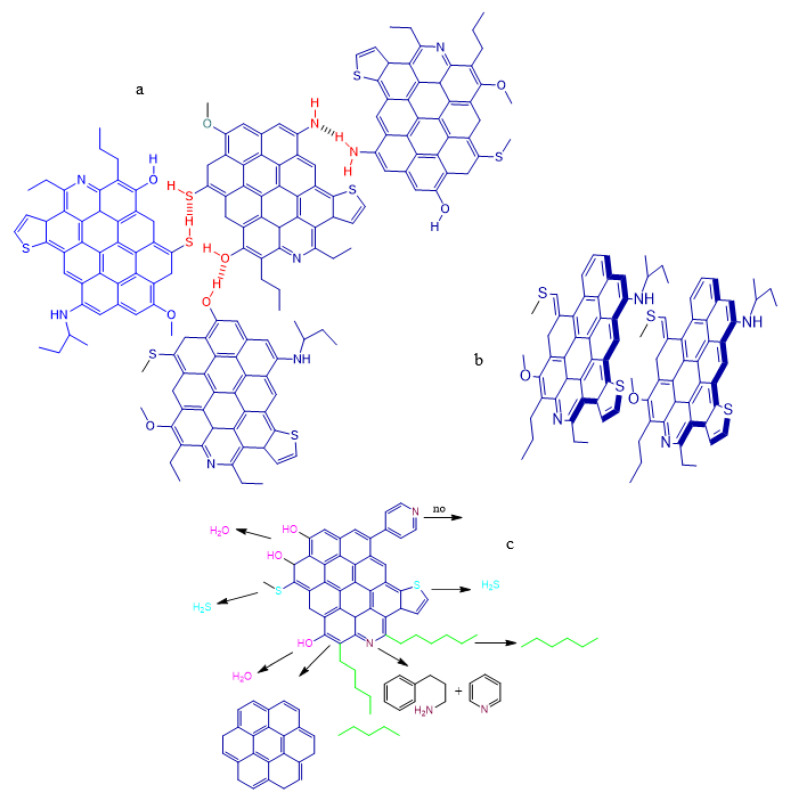
Schematic diagram of catalytic aquathermolysis in heavy oil (**a**) heteroatoms of S, N, and O; (**b**) 3-D network and (**c**) monocyclic compounds.

The high viscosity of heavy oil arises from the interaction of macromolecules, as shown in Figure 14a–c. Heavy oil macromolecules contain a large number of heteroatoms of S, N, and O (Figure 14a); the interaction of hydrogen bonds and π-π stacking between the macromolecules results in the formation of a 3-D network (Figure 14b), resulting in the poor fluidity of heavy oil. The mechanism of the aquathermolysis reaction of heavy oil can be summarized from the reactions of the model compounds used herein. The hydroxyl-containing aromatic ring is more likely to be removed in aquathermolysis. Sulfur-containing ring compounds and aromatic ring-containing compounds are more prone to ring-opening desulfurization, yielding a low-carbon hydrocarbon. Aromatic hydrocarbons with longer alkyl chains are prone to alkyl chain C-C bond breakage. Polycyclic aromatic hydrocarbons with nitrogen are more likely to open up and undergo hydrogenation reactions, while monocyclic compounds react less readily. The mechanism is shown in Figure 14c.

## 5. Conclusions

The synergistic catalysis of an ex situ catalyst and in situ clay in the aquathermolysis of heavy oil was investigated. The effect of aquathermolysis on viscosity reduction is largely influenced by temperature, reaction time, the oil-to-water ratio, and the type of mineral. The introduction of external catalysts and hydrogen donors improves the viscosity reduction efficiency. Under optimal conditions, the viscosity reduction of Oil-water, Oil-water-SB, Oil-water-C-Cr, Oil-water-SBC-Cr, and Oil-water-SBC-Cr-ethanol oil samples reached 46%, 52%, 62%, 68%, and 73%, respectively. The pour point was depressed by 3.0 °C, 5.0 °C, 10.0 °C, 15.0 °C, and 15.0 °C, respectively, showing the synergistic catalysis of the ex situ catalyst and in situ clay in the aquathermolysis of heavy oil. After the catalytic aquathermolysis of heavy oil, the thermogravimetry indicated that the content of the light components increased, with the most pronounced change occurring in the case of the Oil-water-SBC-Cr sample. DSC showed that the wax precipitation point of crude oil and the wax peaks all shifted to the right, with a simultaneous wax content increase. The carbon number distribution shows that the low-carbon number distribution is broader after the heavy oil reaction and their content is greater, with the C15 content increasing the most. The catalyst showed high thermal stability and reusability. After aquathermolysis, the saturation and heteroatom content in the water phase decreased obviously. The findings suggest that this work may be beneficial for related research in the field of oilfield exploration.

## Figures and Tables

**Table 1:** The surface area, pore volume, and pore diameter of SB and SBCCr.

Sample	Pore Diameter/nm	Pore Volume/cm^3^·g^−1^	Specific Surface Area/m^2^·g^−1^
SB	6.2834	0.1155	73.5293
SBCCr	6.0402	0.1099	72.8337

**Table 2:** The effect of the catalysts on the L8401 pour point depression.

Oil Sample	Pour Point/°C
Blank	24.0
OW	21.0
OWSB	19.0
OWCCr	14.0
OWSBCCr	9.0
OWSBCMn	9.5
OWSBCFe	9.5
OWSBCCo	9.0
OWSBCNi	9.5
OWSBCCu	9.0
OWSBCZn	9.5
OWSBCCrE	9.0

**Table 3:** The viscosity of heavy oil after different cycles.

Cycles	Viscosity/mPa·s					Pour Point/°C
	30 °C	40 °C	50 °C	60 °C	70 °C	
1	72,300	25,100	9140	4010	1980	9.0
2	84,700	31,400	10,800	5180	3250	9.0
3	85,500	30,300	11,000	5100	3100	9.5
4	85,400	31,200	11,300	4530	3300	10.0

**Table 4:** The elemental content of heavy oil that has reacted under different conditions.

Oil Sample	C/%	H/%	N/%	S/%
Blank	86.12	10.48	1.51	0.50
OW	88.34	7.50	1.48	0.46
OWSB	84.20	10.09	1.35	0.43
OWSBCCr	81.09	9.95	0.50	0.25
OWSBCCrE	87.43	10.13	1.47	0.43

**Table 5:** New compounds of the phenols under different reaction conditions.

Reactant	Catalysts	Product
phenol, H_2_O	no	Benzene
phenol, H_2_O	SB	
phenol, H_2_O	SBCCr	
phenol, H_2_O, ethanol	SBCCr	

**Table 6:** The new compounds of thiophene under different reaction conditions.

Reactant	Catalysts	Product
thiophene, H_2_O	no	Phenol, toluene
thiophene, H_2_O	SB	
thiophene, H_2_O	SBCCr	
thiophene, H_2_O, ethanol	SBCCr	

**Table 7:** The new compounds of nonylphenol under different reaction conditions.

Reactant	Catalysts	Product
nonylphenol, H_2_O	no	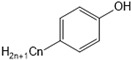 n = 1, 3, 4, 5, 6, 7, 8
nonylphenol, H_2_O	SB	
nonylphenol, H_2_O	SBCCr	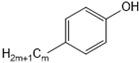 m = 0, 1, 3, 4, 5, 6, 7, 8
nonylphenol, H_2_O, ethanol	SBCCr	

**Table 8:** New compounds of quinoline under different reaction conditions.

Reactant	Catalysts	Product
quinoline, H_2_O	no	Toluene, 3-phenyl-1-propylamine, propylamine
quinoline, H_2_O	SB	Toluene, pyridine
quinoline, H_2_O	SBCCr	
benzothiophene, H_2_O, ethanol	SBCCr	Toluene

**Table 9:** New compounds of benzothiophene under different reaction conditions.

Reactant	Catalysts	Product
benzothiophene, H_2_O	no	Toluene, 2-mercaptotoluene, phenol
benzothiophene, H_2_O	SB	Toluene, phenol
benzothiophene, H_2_O	SBCCr	
benzothiophene, H_2_O, ethanol	SBCCr	Toluene, 2-mercaptotoluene, phenol

**Table 10:** New compounds of pyridine under different reaction conditions.

Reactant	Catalysts	Product
pyridine, H_2_O	no	Pyridine
pyridine, H_2_O	SB	
pyridine, H_2_O	SBCCr	
pyridine, H_2_O, ethanol	SBCCr	

## Data Availability

Not applicable.

## Sample Availability

Samples of the compounds are available from the authors.
